# How Has the COVID-19 Crisis Affected the Academic Stress of University Students? The Role of Teachers and Students

**DOI:** 10.3389/fpsyg.2021.626340

**Published:** 2021-06-01

**Authors:** Jesús de la Fuente, Mónica Pachón-Basallo, Flavia H. Santos, Francisco J. Peralta-Sánchez, María Carmen González-Torres, Raquel Artuch-Garde, Paola V. Paoloni, Martha L. Gaetha

**Affiliations:** ^1^School of Education and Psychology, University of Navarra, Pamplona, Spain; ^2^School of Psychology, University of Almería, Almería, Spain; ^3^School of Psychology, University College Dublin, Dublin, Ireland; ^4^School of Health Sciences, Public University of Navarra, Pamplona, Spain; ^5^School of Education, Universidad de Río Cuarto, Córdoba, Argentina; ^6^School of Social Sciences, University of Puebla, Puebla, Mexico

**Keywords:** COVID-19, academic stress, achievement emotions, engagement-burnout, gender, undergraduates students, teaching–learning

## Abstract

The effects of the COVID-19 pandemic have required substantial adjustments in terms of university teaching–learning processes. The aim of this study was to verify whether there were significant differences between the academic year of 2020 and the two preceding years in factors and symptoms and stress. A total of 642 university students (ages 18–25 years) participated by filling out validated self-reports during the months from March to August 2020. Using an ex post facto design, SEM analyses and simple and multiple ANOVAs were performed. Structural results showed that stress factors from the teaching process had a predictive value for the learning process, emotions, and academic burnout, and being a man was a factor predicting negative emotion. In a similar way, inferential results revealed no significant effect of academic year but did show an effect of gender on stress experiences during the pandemic. Aside from certain specific aspects, there was no significant global effect of the year 2020 on factors and symptoms of stress. The results showed that studying in the year of the COVID-19 outbreak did not have a significant effect on stress triggered by the teaching process. From these results, we draw implications for specific guidance interventions with university teachers and students.

## Introduction

Numerous health-related studies (Cancello et al., 2020; Kaushal et al., 2020; Kannampallil et al., 2020) and research topics have been set in motion due to the recent COVID-19 pandemic. In the same way, factors of well-being and achievement emotions are making a strong appearance in psychoeducational research, particularly the consequences of stressful events, because of their potential psychological impact on university teaching and learning processes (Salanova et al., 2005; Durand-Bush et al., 2015; Berry, 2020).

### Potential Factors of Academic Stress During the Teaching–Learning Process at University

The rapid expansion of the coronavirus pandemic has disrupted life for persons, states, and institutions worldwide. Feelings of great uncertainty and anxiety have been triggered (Kowal et al., 2020; Ramos-Lira et al., 2020). This situation has posed a real challenge and a dramatic change for the university in general, and for professors and students (Kecojevic et al., 2020). Academic life was abruptly confined to the home, and the ordinary activity of the university, with its face-to-face teaching and learning, has had to be substituted by online teaching and remote learning (Sahu, 2020). With this scenario, it is reasonable to expect that university life has become even more stressful than usual for many students (Ahern and Norris, 2011; Denovan and Macaskill, 2017; Szulevicz et al., 2019; American College Health Association (ACHA), 2020; Hasan et al., 2020; Song et al., 2020). This perceived stress would then have an ongoing influence on their emotions, on how they engage in the learning process, and their psychological well-being (Capone et al., 2020; Kecojevic et al., 2020; Rodríguez-Hidalgo et al., 2020).

Prior research has identified several factors of academic stress pertaining to the teaching–learning process (de la Fuente et al., 2020c; Karaman et al., 2017), that is, factors that may provoke stress in students (de la Fuente et al., 2011). Stress factors pertaining to the *teaching process* include maladjusted teaching methodology, poor classroom climate, and irrelevant content; factors related to the *learning process* include an excess of learning activities (perceived as a heavy workload), student presentations in class, and an assessment system that induces lack of control over one’s achievement (González-Cabanach et al., 2008, 2016, 2017). The specific causes of these effects, yet to be evaluated, fall within the scope of educational psychology and its study of academic stress (de la Fuente et al., 2017).

### The Teaching Process as a Factor of Academic Stress

Direct and indirect changes in the academic life of universities, brought about by the COVID-19 pandemic, have attracted research interest. We are all aware of the numerous adjustments that have been made in a short period of time, transitioning from face-to-face teaching systems to distance learning or combination formats, as well as adjustments made to university syllabi, learning activities, online exams, and adaptations in class attendance. Ultimately, the COVID-19 experience has become a stress test—to borrow a concept from banking—for our university system (Hasan et al., 2020; Song et al., 2020). *Regulatory teaching* refers to a good teaching style, in that it favors a good learning process. There is ample evidence for teaching style being a predictor of student engagement, motivation, and well-being, and it is a *buffering* factor against academic stress (Codina et al., 2020; Dash et al., 2020). Teacher profile, referring to their emotions and their own motivations, has also been found to positively or negatively affect the learning process (Moè and Katz, 2020a,b; Vermote et al., 2020).

The *Self- vs Externally-Regulated Learning* (SRL vs ERL) *Theory* (2017) is a complementary perspective and a valid heuristic for analyzing this reality. Reports of previous evidence have already revealed effects in this direction, in different motivational variables (de la Fuente et al., 2017), positive and negative emotionality (de la Fuente et al., 2020b), coping strategies (de la Fuente et al., 2020a), and factors and responses to academic stress (de la Fuente et al., 2020c). Based on the theory’s assumptions of regulation, nonregulation, and dysregulation (internal and external), the following types of teaching–learning contexts may be described:

1) *Regulatory teaching–learning.* This is the case where teachers have properly planned and designed the teaching–learning process, including a range of technical support that allows the process to proceed adequately and be fitted to the new situation with minimal planning changes. In this scenario, students are less likely to show stress symptoms, negative emotionality, and burnout, and motivational behaviors of engagement can be maintained.
2) *Nonregulatory teaching–learning.* In this case, where teachers have prepared only face-to-face learning without the use of online technologies, their planning is not compatible with the new situation of online teaching. Clear teaching-learning guidelines for the new situation do not exist. Students feel uncertain about the way forward, and external regulation is lacking, thus increasing the likelihood of stress symptoms, negative emotionality, and a certain degree of burnout.
3) *Dysregulatory teaching–learning.* In such cases, teachers follow an irregular pattern; previous planning is lacking, and they make arbitrary decisions about teaching and learning in the new situation. Assessment criteria undergo changes and unexpected new activities are incorporated. Consequently, students feel overwhelmed by the demands, are plagued with uncertainty and negative emotionality, and show greater levels of burnout.

### Student Characteristics as a Factor of Academic Stress: Gender Differences

The potential psychological and academic impact of confinement during the COVID-19 pandemic, students’ management of stress, and a possible gender modulation, among other factors, are subjects of growing interest in the most recent psychoeducational research (Ahuja et al., 2020; Cao et al., 2020; Harutyunyan et al., 2020; Rogowska et al., 2020; Rodríguez et al., 2020; Ribeiro et al., 2021). There are certain disparities in the research regarding increased academic stress during the COVID-19 outbreak (Capone et al., 2020; Rogowska et al., 2020), its repercussions on the well-being of students from different cultures (Rogowska et al., 2020) and the role of gender differences (Pomerantz et al., 2002; Harutyunyan et al., 2020; Rogowska et al., 2020). Many research studies indicate that, both in the general population and the university population, women present higher levels of stress (Loureiro et al., 2008; Cabanach et al., 2009). In prior research reports, however, there is a lack of agreement in this regard (Matheny et al., 2008; Ahern and Norris, 2011; Rodríguez et al., 2020), so the contribution of this demographic variable requires further exploration. Among the main academic stressors that students refer to are deficiencies in teaching methodology, excessive workload, public speaking (presentations), exams, and poor social relations within the academic context.

All the foregoing aspects seem to be modulated by the gender variable, with ample supporting evidence already (Richardson and King, 1991; Davis, 1995; Sadler-Smith, 1996; Andreou et al., 2006; Else-Quest et al., 2006; Brougham et al., 2009). Recent research has shown that being a woman is associated with and is a determinant of higher anxiety levels in university students (Gao et al., 2020), but it is also associated with higher self-regulation (Duckworth and Seligman, 2006), academic behavioral confidence (de la Fuente et al., 2013a, b; Sander and de la Fuente, 2020), engagement, resilience, and academic achievement (Durand-Bush et al., 2015). Moreover, men are associated with and are a determinant of procrastination and poorer achievement (Garzón-Umerenkova et al., 2018). For these reasons, the present investigation analyzes whether this usual tendency has been intensified as a consequence of the new context we are facing.

### Achievement Emotions as a Correlate of Academic Well-Being or Discomfort

Academic obstacles related to learning and teaching (or academic stress) affect one’s emotionality toward academic tasks. There is plenty of recent evidence that achievement emotions are a significant correlate of academic well-being and of the degree of satisfaction with the academic experience at university (Garett et al., 2017; Frenzel et al., 2018). When this emotionality is positive, it is reasonable to infer that positive emotions exist during the process, such as enjoyment, pride, satisfaction. Negative emotions, such as boredom, anger, anxiety, or hopelessness, suggest the opposite: that there are maladjustment issues while learning (Pekrun et al., 2005; Vermunt, 2007).

Certain studies highlight differences in the associations between academic stress and academic well-being, burnout, and engagement (Extremera et al., 2007). Pekrun et al. (2002) consider that the study of affect in educational psychology should address the full range of students’ affective experiences, negative as well as positive. In recent years, the control-value theory of achievement emotions (CVTAE) is being used to examine how emotions shape student engagement and learning (Linnenbrink-García and Pekrun, 2011; Kahu et al., 2015; Gelabert-Carulla and Muntaner-Mas, 2017; Burr and Dallaghan, 2019; de la Fuente et al., 2020b).

### The Motivational State of Engagement Burnout in University Students

The phenomena of *burnout* and *engagement* have been analyzed profusely in the organizational context (Maslach et al., 1996), but in the past two decades they are also the object of study in the academic context (Christenson et al., 2012) and at the university level (Martínez et al., 2002; Schaufeli et al., 2002; Yang, 2004; Salanova et al., 2005; Mostert et al., 2007; Zhang et al., 2007; Casuso-Holgado, 2011; Friedman, 2014).

The stress experienced by the student is seen as an important predictor of their motivational state of burnout/engagement (Salanova et al., 2010). Differences in motivational state may depend on the subject’s dispositional variables, such as self-efficacy (Salanova et al., 2011) and emotional intelligence (Durán et al., 2006), on sociodemographic variables (sex and age), and on educational variables (teaching methods and guidance) (Lekwa et al., 2018).

The motivational state of academic engagement-burnout has also been analyzed, where engagement was observed to be directly proportionate to the degree of students’ self-regulation and a regulatory teaching process. Higher levels of self-regulation mean a stronger motivational state of engagement and less burnout; lower levels show the opposite (de la Fuente et al., 2017).

### Aims and Hypotheses

Based on the above, the *aims* of this study were as follows: (1) to analyze whether the students’ perception of teaching and learning stress factors predicted significant changes in achievement emotions and motivational state of engagement-burnout and whether the academic year and gender could also predict these emotional changes; (2) to inferentially analyze the specific causal effects that the *academic year* and *gender* had on stress factors originating in the teaching–learning process, on negative emotions, and on the state of engagement-burnout of undergraduate students.

We established the following *hypotheses*. (1) The perception of stressors in teaching will positively and significantly predict learning stressors; and these will, in turn, predict negative emotions, as well as students’ state of engagement-burnout. Additionally, this relationship will be predicted by the COVID-19 academic year and by gender. (2) The *year* and *gender* factors will have a significant main effect on the level of the teaching-learning factors of stress, negative emotions, and the state of engagement-burnout. These results would be differentiated according to gender, based on prior evidence, with men showing more emotional decline toward burnout and women showing greater engagement and greater test anxiety.

## Materials and Methods

### Participants

A total of 642 university students (between the ages of 18 and 25) participated in this study. Of these, 201 students participated in 2018, 168 students in 2019, and 305 in 2020. The mean age was 20.42 years (SD = 5.8), and the age range was 19–25 years. Participation was anonymous and voluntary. Lecturers from various departments were invited to participate, and those who agreed then extended the invitation to their students. Participating lecturers and students were awarded a Certificate of Participation. Online questionnaires were applied to assess each specific teaching–learning process. The groups of participating students were different and from different academic subjects. All of them were studying for Degrees in Psychology and Education. Group equivalence was checked using the relevant statistical analyses (see section “Data Analyses”).

### Instruments

#### Factors of Stress

The Academic Stress Questionnaire, CEA/ASQ (González-Cabanach et al., 2008). First, the internal structure of the scale was analyzed. For this purpose, we used confirmatory factor analysis (CFA) with the entire data set from our sample. The default model showed good fit [chi-square or CMIN = 66.457, df = 13, *p* < 0.001; relative chi-square or CMIN/df = 5.11; SRMR = 0.075, CFI = 0.935, TLI = 0.961, IFI = 0.947, RFI = 0.965, NFI = 0.947, RMSEA = 0.057, HOELTER = 0.430 (*p <* 0.05) and 0.532 (*p <* 01)]. The proposed model contained 53 items with a seven-factor structure having two dimensions, where one factor differs from the original version. The resulting dimensions and factors were: (1) Dimension of Stress in Learning: Heavy Workload (Factor 2), Lack of Control over Achievement (F3), Social climate (Factor 5), and Test Anxiety (Factor 7); (2) Dimension of Stress in Teaching: Methodology difficulties (Factor 1), Public speaking (Factor 4); Content lacking value (Factor 6). Overall reliability, Alpha = 0.961; part 1, Alpha = 0.932, part 2, Alpha = 0.946, in this study. Some examples of items are as follows: “I get nervous or tense… when they ask me questions in class” and “It worries me…that the subjects we are studying are of little interest.”

#### Achievement Emotions in the Study Situation

We measured achievement emotions with a validated Spanish version (Paoloni et al., 2014; de la Fuente, 2015a, b; Paoloni, 2015) of the Achievement Emotions Questionnaire (Pekrun et al., 2005), which had adequate reliability and construct validity values. The questionnaire was one of the outcomes of a qualitative and quantitative research program that analyzed student emotions within academic achievement situations. Several discrete emotions are measured, as they appear in the three primary situations of academic achievement: class time, study time, and doing tests and exams. Each of the three sections of the questionnaire corresponds to one of these situations, respectively. In total, 80 items in the class-related emotions scale (CRE) measure the following eight emotions as they occur during class: enjoyment, hope, pride, anger, anxiety, shame, hopelessness, and boredom. The learning-related emotions scale (LRE) contains 75 items that measure the same eight emotions in study situations. The test emotions scale (TE) measures these emotions in testing situations, using 77 items. Each of the scales contains three subscales that measure emotions appearing before, during, or after the corresponding situation under assessment. Trait achievement emotions are assessed, that is, the student’s typical emotional reactions to each type of achievement situation. Instructions for the AEQ can be modified, as needed, to measure emotions experienced in a particular class subject (course-specific emotions) or in specific situations at specific moments (state achievement emotions). Example items include the following: emotions at the start of study (“I have an optimistic view toward studying”); emotions during study time (“Because I’m bored, I get tired sitting at my desk”), and emotions when finishing study (“I am so happy about the progress I made that I am motivated to continue studying”). Internal consistency of the class situation scale is good (Alpha = 0.904; Part 1, Alpha = 0.803; Part 2, Alpha = 0.853). Internal consistency of the study situation scale is adequate (Alpha = 0.939; Part 1, Alpha = 0.880, Part 2, Alpha = 0.864). Internal consistency of the testing situation scale is sufficient (Alpha = 0.913; Part 1, Alpha = 0.870, Part 2, Alpha = 0.864). Students report their own emotions according to type (positive vs. negative) and intensity (from 1 = none to 5 = very strong). Examples of items are as follows: “BEFORE STUDYING … I get so nervous that I don’t even want to begin to study”; “DURING STUDY… I worry whether I’m able to cope with all my work”; and “AFTER STUDYING… I’m proud of myself.”

#### Engagement Burnout

A validated Spanish version of the *Utrecht Work Engagement Scale for Students* (Schaufeli et al., 2002) was used to assess engagement in our study sample. The model obtained good fit indices in this sample. We confirmed multidimensionality of the scale and metric invariance in our samples (Chi-square = 792.526, df = 74, *p <* 0.001; CFI = 0.954, TLI = 0.976, IFI = 0.954, TLI = 0.979, and CFI = 0.973; RMSEA = 0.083; HOELTER = 153, *p <* 0.05; 170 *p <* 0.01). Cronbach alpha in this sample was.900 (14 items), with 0.856 (7 items) and 0.786 (7 items) for the two parts, respectively. A validated Spanish version of the *Burnout Scale for Students* (Schaufeli et al., 2002) was used to assess burnout. Psychometric properties for this version were satisfactory in students from Spain. The model obtained good fit indices in this sample. We confirmed multidimensionality of the scale and metric invariance in our samples (Chi Square = 767.885, df = 87, *p* < 0.001; CFI = 0.956, TLI = 0.964, IFI = 0.951, TLI = 0.951, and CFI = 0.953; RMSEA = 0.071; HOELTER = 224, *p <* 0.05; 246 *p <* 0.01). Cronbach’s alpha for this sample was Alpha = 0.874 (15 items), with Part 1, Alpha = 0.853 (8 items) and Part 2, Alpha = 0.793 (7 items) for the two parts, respectively. Examples of items are as follows: “I feel happy when I am studying intensively” and “I doubt the significance of my studies.”

### Procedure

Researchers from the present project were asked to invite students from their university to complete the questionnaires mentioned above during the months from March to August 2020. Samples of these questionnaires had previously been collected during the same months of 2018 and 2019. We followed the same protocol that was established and approved by the ethics committee of the University of Navarra (ref. 2018.170). Questionnaires were completed online, on a voluntary basis, outside of class time. An automated tool (www.inetas.org) had been designed for this purpose (de la Fuente et al., 2015). Both the students and the teachers involved were offered certification of their participation in the Research Project.

### Data Analyses

The research design was ex post facto, non-linear, and inferential using a non-probabilistic convenience sample. To test the hypotheses posed, we carried out previous analyses using Levene’s test, in order to ensure equality of variances of errors.

1) For the *structural predictive hypotheses*, confirmatory factor analysis was tested with a Structural Equation Model (SEM) in this sample. Data were aggregated using the determination of factors obtained in the corresponding previous exploratory and confirmatory factor analyses (not summationally) in order to avoid false positives. We assessed model fit by first examining the ratio of chi-square to degrees of freedom, SRMR, then the Comparative Fit Index (CFI), Normed Fit Index (NFI), Incremental Fit Index (IFI), and Relative Fit Index (RFI). Ideally, these should all be greater than 0.90. Sample size adequacy was checked using the Hoelter Index (Tabachnick and Fidell, 2001). AMOS (v.22) was used for the latter analyses.
2) For the *inferential hypotheses*, we carried out ANOVAs and MANOVAs, 3 (year = 2018, 2019, and 2020) × 2 (gender = Men and Women), using Pillai’s index. The software package SPSS v. 25 (New York) was used for these analyses.

## Results

### Structural Prediction Model

Several models were tested in order to validate the hypothesis of the year × gender effect. *Model 1* included the effect of both variables (teaching and learning factors of stress) on positive vs. negative emotions, as well as engagement vs. burnout, but it was not considered positive. *Model 2* tested the effect of both variables (teaching and learning factors of stress) only on negative emotions and burnout, with more acceptable, but still insufficient, values. *Model 3* tested the effect of the academic variables (teaching and learning factors of stress, year, and gender) on stress factors, negative emotions, and burnout, achieving acceptable values. The values of each model are presented in Table 1.

**TABLE 1:** Statistical values of the models tested.

Model	Chi/df *p* <	Ch/df	SRMR	NFI	RFI	IFI	TLI	CFI	RMSEA	HOELT
1.	1657.529/146***	11.353	0.15	0.884	0.796	0.855	0.811	0.855	0.082	177
2.	1560.686/145***	10.763	0.12	0.853	0.807	0.865	0.822	0.864	0.079	187
3.	666.764/100***	6.667	0.07	0.928	0.939	0.929	0.953	0.929	0.075	218

***p < .001.

**FIGURE 1:**
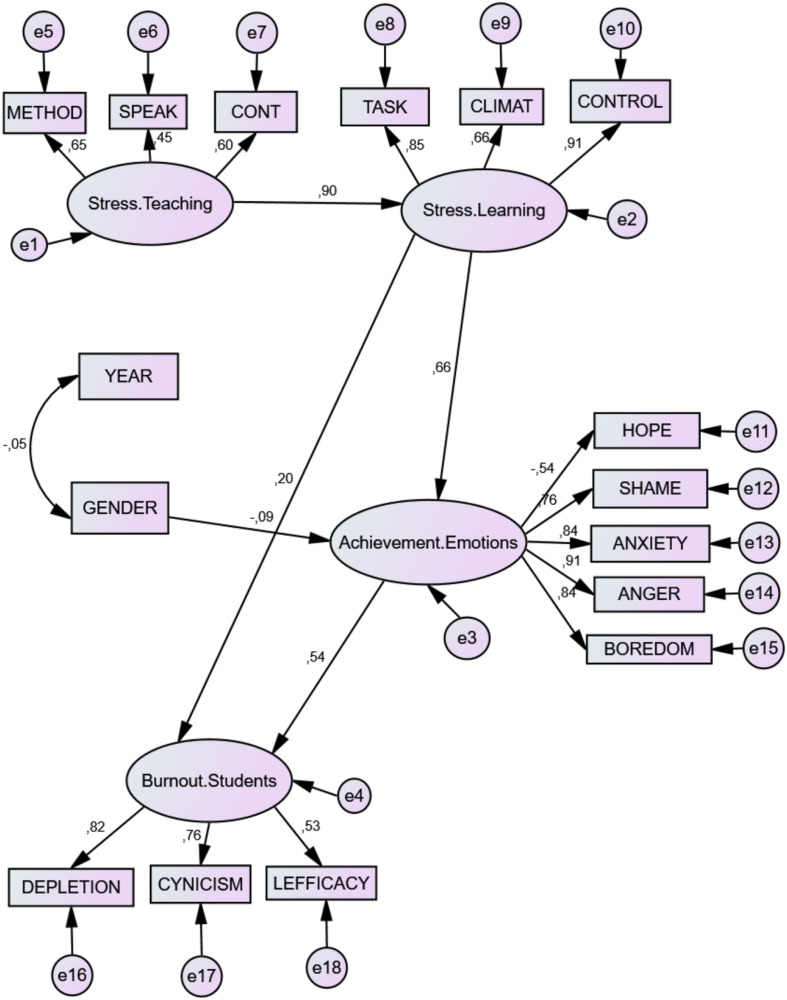
Structural predictive model. Stress.Teaching = Stress Factors in Teaching process; Stress.Learning = Stress Factors in Learning process; Achievement.Emotions = achievement emotions during study; Burnout.Students = Burnout; METHOD = methodological difficulties; SPEAK = speaking in public; CONT = value of the content; TASK = heavy workload; CLIMAT = negative social climate; CONTROL = lack of control over achievement; HOPE = Hope.Study; ANXIETY = Anxiety.Study; ANGER = Anger.Study; SHAME = Shame.Study; BOREDOM = Boredom.Study; DEPLETION = Exhaustion; CYNICISM = cynicism; LEFFICACY = Lack of efficacy. Gender (1 = Men; 2 = Women).

Direct effects were found, showing that gender (Men = 1 and Women = 2) negatively predicted negative emotions during the study (*B* = –0.90). Stress factors of teaching were also found to positively predict the stress factors of learning (*B* = 0.943). Stress in learning positively predicted negative emotions (*B* = 0.692) and to a lesser degree burnout (*B* = 0.215). However, negative emotions while studying strongly predicted Burnout (*B* = 0.512). See Table 2 and Figure 1.

**TABLE 2:** Standardized Direct Effects of prediction.

	Gender	Stress factors of Teaching	Stress factors of Learning	Negative Emotions	Burnout
Stress Teaching					
Stress Learning		0.943			
Negat. Emotions	–0.90		0.692		
Burnout			0.215	0.512	
Value cont.		0.586			
Negative climate		0.679			
Method diffic.		0.622			
Lack of control			0.912		
Public speaking			0.484		
Heavy workload			0.852		
Hopel.Study				0.926	
Shame.Study				0.846	
Boredom.Study				0.785	
Anger.Study				0.863	
Anxiety.Study				0.869	
Depletion					0.834
Cynicism					0.799
Low Efficacy					0.551

*Stress Teaching = Stress factors in Teaching; Stress Learning = Stress factors in Learning); Negat. Emotions = Negative emotions while learning; Burnout = Burnout; Value cont. = Value of the content; Negative climate = negative social climate; Method diffic. = methodological difficulties; Lack of control = lack of control over achievement; Public speaking = speaking in public (presentations); Heavy workload = heavy workload; Hopel.Study = Hopelessness.Study; Shame.Study = Shame.Study; Boredom.Study = Boredom.Study; Anger.Study = Anger.Study; Anxiety.Study = Anxiety.Study; Depletion.Study = exhaustion; Cynicism = cynicism; Low Efficacy = Lack of efficacy.*

Indirect prediction effects were of particular interest. Gender (M = 1 and F = 2) was a negative but less consistent predictor of burnout (–0.046), of negative emotions while learning, and of the factors of burnout. Stress factors in Teaching appeared as positive indirect predictors—with greater consistency—of negative emotions (*B* = 0.652) and burnout (*B* = 0.536). Finally, stress factors while learning appeared as indirect predictors of burnout (*B* = 0.354). See Table 3.

**TABLE 3:** Standardized Indirect Effects of prediction.

	Gender	Stress factors of Teaching	Stress factors of Learning	Negative Emotions	Burnout
Stress Teaching					
Stress Learning					
Negat. Emotions		0.652			
Burnout	–0.046	0.536	0.354		
Value cont.					
Negative climate					
Method diffic.					
Lack of control		0.860			
Public speaking		0.456			
Heavy workload		0.804			
Hopel.Study	–0.083	0.604	0.640		
Shame.Study	–0.076	0.552	0.585		
Boredom.Study	–0.071	0.512	0.543		
Anger.Study	–0.078	0.563	0.597		
Anxiety.Study	–0.078	0.567	0.601		
Depletion	–0.038	0.447	0.474	0.427	
Cynicism	–0.037	0.428	0.454	0.409	
Low Efficacy	–0.025	0.295	0.313	0.282	

*Stress Teaching = Stress factors in Teaching; Stress Learning = Stress factors in Learning); Negat. Emotions = Negative emotions while learning; Burnout = Burnout; Value cont. = Value of the content; Negative climate = negative social climate; Method diffic. = methodological difficulties; Lack of control = lack of control over achievement; Public speaking = speaking in public (presentations); Heavy workload = heavy workload; Hopel.Study = Hopelessness.Study; Shame.Study = Shame.Study; Boredom.Study = Boredom.Study; Anger.Study = Anger.Study; Anxiety.Study = Anxiety.Study; Depletion.Study = exhaustion; Cynicism = cynicism; Low Efficacy = Lack of efficacy.*

### Effect of Academic Year and Gender on Factors of Academic Stress

#### Effect of Year and Gender on Total Level of Factors of Academic Stress

The previous analyses showed an adequate level of homogeneity of variance [*Levene Test* (5,668) = 0.884, *p <* 0.519]. The ANOVA revealed a single main effect of *gender* on the level of total *stress factors*, tending toward women (that is, women showed higher levels of total academic stress factors) and no significant interaction effect. Note that the most important effect of gender was found in stressors in the learning process. See values in Table 4.

**TABLE 4:** Descriptive values of the different levels of Stress Factors: M(SD) (*n* = 674).

Year	2018				2019			2020			
Gender	M	F	T	M	F	T	M	F	T		
(n = )	(39)	(162)	(201)	(47)	(121)	(168)	(72)	(233)	(305)	Effects, *F* (Pillais test)	Post
*Total stress*	2.85(0.67)	2.94(0.70)	2.29(0.69)	2.81(0.76)	3.07(0.67)	3.00(0.70)	2.76(0.63)	3.11(0.66)	3.03(0.67)	*G*, *F*(1,668) = 13.089***, *r*^2^ = 0.019, *power* = 0.91	F > M
Teaching stress	2.80(0.67)	2.88(0.73)	2.86(0.71)	2.85(0.84)	3.05(0.73)	3.00(0.77)	2.77(0.62)	3.03(0.70)	2.97(0.70)	*G, F*(2,636) = 7.949***, *r*^2^ = 0.024; *power* = 0.995; *G*, *F*(1,637) = 6.538**, *r*^2^ = 0.010; *power* = 0.72	F > M
Learning Stress	2.84(0.82)	2.94(0.78)	2.92(0.79)	2.72(0.85)	3.01(0.72)	2.97(0.73)	2.64(0.74)	3.13(0.74)	3.02(0.77)	*G, F*(1,636) = 15.923***, *r*^2^ = 0.024; *power* = 0.979; YxG, *F*(2,637) = 2.536, *p <* 0.08, *r*^2^ = 0.008; *powe*r = 0.508	F > M
Methodology Difficulties	3.35(0.73)	3.49(0.80)	3.46(0.79)	2.39(0.88)	3.52(0.89)	3.48(0.88)	3.31(0.79)	3.56(0.87)	3.56(0.84)	G, *F*(6,632) = 3,478***, *r*^2^ = 0.032; *power* = 0.948; G, *F*(1,637) = 7.349**, *r*^2^ = 0.011; *powe*r = 0.742	F > M
Heavy Workload	2.72(0.83)	2.84(0.90)	2.82(0.89)	2.81(0.96)	2.97(0.82)	2.92(0.96)	2.67(3.19)	3.19(0.88)	3.07(0.97)	G, *F*(1,637) = 10,050***, *r*^2^ = 0.016; *power* = 0.886; Y x G, *F*(2,637) = 2.678, *p <* 0.06, *r*^2^ = 0.008; *power* = 0.571	F > M
Public speaking	3.22(1.15)	3.28(1.07)	3.27(1.09)	3.32(1.17)	3.17(1.22)	3.22(1.20)	2.68(1.17)	3.41(1.16)	3.23(1.20)	G, *F*(1,637) = 16.456***, *r*^2^ = 0.025; *power* = 0.482; Y x G, *F*(2,637) = 2.548, *p <* 0.07, *r*^2^ = 0.008; *power* = 0.510	F > M
Lack of control over achievement	2.57(0.83)	2.66(0.81)	2.65(0.82)	2.54(0.91)	2.68(0.71)	2.64(0.72)	2.45(0.72)	2.74(0.80)	2.67(0.79)	G, *F*(1,637) = 4.991***, *r*^2^ = 0.008; *power* = 0.607	F > M
*Achievement Emotions*										G, *F*(8,658) = 3.965***, *r*^2^ = 0.046; *power* = 0.992	
Boredom in Study	2.48(0.83)	2.31(0.82)	2.34(0.83)	2.50(0.99)	2.16(0.89)	2.27(0.94)	2.35(0.83)	2.14(0.88)	2.19(0.87)	G, *F*(1,665) = 8.572***, *r*^2^ = 0.013; *power* = 0.832	M > F
Anxiety in Study	2.47(0.74)	2.66(0.72)	2.62(0.73)	2.59(0.74)	2.40(0.74)	2.46(0.74)	2.41(0.75)	2.55(0.71)	2.51(0.72)	Y x G, *F*(1,665) = 3.348*, *r*^2^ = 0.010; power = 0.632	F > M
*Engagement-Burnout*										G, *F*(2,766) = 4.841***, *r*^2^ = 0.012; *power* = 0.800	
Engagement	3.14(0.75)	3.42(0.68)	3.37(0.70)	3.18(0.65)	3.43(0.76)	3.31(0.71)	3.24(0.70)	3.40(0.74)	3.26(0.76)	G, *F*(1,777) = 9,333***, *r*^2^ = 0.012; *power* = 0.862	F > M
Burnout	2.50(0.68)	2.34(0.75)	2.39(0.68)	2.51(0.69)	2.24(0.65)	2.31(0.62)	2.39(0.68)	2.37(0.67)	2.37(0.69)	G, *F*(1,777) = 6,140***, *r*^2^ = 0.008; *power* = 0.697	M > F
Factors										G, *F*(6,772) = 3.698***, *r*^2^ = 0.028; *power* = 0.961	
Cynicism	2.38(0.92)	2.18(0.90)	2.21(0.90)	2.28(1.00)	2.14(0.99)	2.19(1.10)	2.53(0.88)	2.24(0.94)	2.32(0.94)	G, *F*(1,777) = 11.401***, *r*^2^ = 0.014; *power* = 0.921	M > F
Dedication	3.71(0.77)	3.81(0.68)	3.79(0.70)	3.69(0.87)	3.79(0.83)	3.76(0.84)	3.54(0.84)	3.85(0.81)	3.76(0.83)	G, *F*(1,777) = 5,285*, *r*^2^ = 0.012; *power* = 0.800	F > M
Absorption	2.97(0.92)	3.27(0.83)	3.21(0.85)	2.92(0.70)	3.18(0.95)	3.11(0.92)	3.14(0.88)	3.08(.099)	3.09(0.96)	G, *F*(1,777) = 4.052*, *r*^2^ = 0.005; *power* = 0.520	F > M

G = Gender effect; Y = Year effect; Y x G = Year x Gender effect. ***p < 0.001. **p < 0.01. *p < 0.05.

#### Effect of Year and Gender on the Factors of Academic Stress Pertaining to the Teaching–Learning Process

The previous analyses showed an adequate level of homogeneity of variance, both for stress factors of teaching [*Levene Test* (5,637) = 1.537, *p <* 0.176] and stress factors of learning [*Levene Test* (5,637) = 0.592, *p <* 0.706]. Regarding the MANOVA, there was a single significant main effect, referring to *gender*, with a higher level of stress factors for women. The partial effects showed an effect of gender in stress factors of the *teaching process*, and of the *learning process*. Finally, there was a marginally significant interaction effect of *year x gender* for stress factors of the *learning process.* See raw descriptive values and effects in Table 4.

#### Effect of Year and Gender on Specific Stress Factors Pertaining to the Teaching–Learning Process

The previous analyses showed an adequate level of homogeneity of variance, both for stress factors of teaching [*Levene Test* (5,637) = 1,537, *p <* 0.176] and stress factors of learning [*Levene Test*(5,637) = 0.592, *p <* 0.706]. Regarding the MANOVA, there was a single significant main effect referring to *gender*, with a higher level of specific stress factors for women. The partial effects showed an effect of *gender* on the following stress factors: in the teaching process, these were *methodological difficulties* and *heavy workload* (this factor with higher power), and in the learning process, these were *public speaking* and *lack of control over achievement*. Finally, there were two, marginally significant, *year x gender* interaction effects, for stress factors of the teaching process: *heavy workload* and *public speaking*. See the descriptive values and effects in Table 4.

### Effect of Academic Year and Gender on Achievement Emotions

#### Effect on Total Positive and Negative Emotions During Study

The Levene test, based on the mean, showed an absence of significant differences in errors of variance, for both positive emotions [*Levene test* (5,665) = 0.911; *p* < 0.437] and negative emotions [*Levene Test* (5,665) = 0.527; *p* < 0.756]. There was no significant main or partial effect of year, gender, or year × gender on total achievement emotions during study.

#### Effect on Specific Positive and Negative Emotions During Study

The Levene test, based on the mean, showed an absence of significant differences in errors of variance, for all dependent variables analyzed. There was a significant main effect of *gender* on total achievement emotions. The partial effects showed an effect of gender on the emotion of boredom, tending toward men, and academic *year x gender* interaction on the response to anxiety, tending toward women. See Table 4.

### Effect of Academic Year and Gender on Engagement-Burnout

The analyses of the variance of error revealed no significant differences, whether for *Engagement* [Levene test (5.745) = 0.838, *p <* 0.523)] or *burnout* [Levene test (5.745) = 0.168, *p <* 0.974)]. The MANOVA revealed a significant main effect of *gender* on total engagement-burnout—an effect that was partially maintained for each total score of the motivational states, *engagement* (in favor of women), and *burnout* (in favor of men).

A significant main effect of *gender* also appeared in the set of all engagement-burnout factors. The partial effects showed a significant partial effect of gender on *cynicism*, tending toward men, as well as effects on *dedication* and *absorption*, tending toward women. Raw values are shown in Table 4.

## Discussion

The *first aim* of the present research specifically examines the predictive effect between university students’ perception of academic stress (induced from the teaching and learning process), achievement emotions, and motivational state of engagement-burnout during the period of the COVID-19 outbreak and in the two previous academic years, taking into account gender differences (*Hypothesis 1*).

In this case, the hypotheses were partially fulfilled. Firstly, consistent positive predictive relationships were found between stress factors during the teaching process (methodological difficulties, and the lack of content value, mainly) and stress factors in the learning process (excess of activities and lack of control over achievement, mainly). In turn, these variables predicted the absence of positive emotions and the presence of negative emotions, as well as academic burnout. These initial results are intrinsically interesting because they show the potential stressful effect that the teaching process had on the learning process. Research on teaching styles has shown that teaching style can be a stress inducer (Dash et al., 2020). This evidence also provides empirical support for the hypothetical relationship between the stress factors that arise from the teaching process, and their effect on learning. A higher perceived level of academic stress (greater negative emotionality and level of burnout) in the learning process is thereby shown to be predicted by greater factors of stress in the teaching process (Moè and Katz, 2020a,b). By contrast, concerning the predictive value of the academic year, it was found that despite the COVID-19 outbreak, no statistical effect was found supporting such relationships. Therefore, the academic year was not a predictor of changes in the factors investigated. This invariance of results could be explained by a continuity in the teaching style of lecturers, which seems to have operated as a protective factor buffering against the experience of stress during the COVID-19 pandemic (Codina et al., 2020). However, gender significantly predicted differences in positive and negative emotionality as well as in engagement burnout.

The *second aim* was fulfilled because the inferential results corroborated, in greater detail, the initial predictive analysis. Hypothesis 2 was partially fulfilled since the year examined (COVID-19 context) did not have a sufficiently significant impact on the variables, while the gender factor appeared to show significant statistical power to determine the value of the dependent variables analyzed. First, although there was no significant general effect of academic year (during the COVID-19 outbreak), a significant increase in certain stress factors of teaching appeared during the year 2020 (COVID-19 context), such as methodological difficulties and heavy workload. Although this effect has been less analyzed, it was to be expected, concurring with other prior evidence in mental health contexts (Chi et al., 2020). However, the greatest statistical effect was from gender, with such strength that it minimized the effect of the context year that we were analyzing.

### Teaching Style as a Modulator of Students’ Emotions and Academic Stress at University

Derived from the general theoretical model of SRL vs ERL (de la Fuente, 2017), characteristics of teaching are of interest, given that the teacher’s regulatory capacity (external regulation) comes into play in making the necessary adjustments within the COVID-19 situation. Remember that *regulatory teaching* (as effective teaching) is designed in a way that clearly and precisely selects and establishes the times, learning activities, content, technology resources, demands, and assessment systems in order to help students learn in a regulated manner (Roehrig et al., 2012), similar to an *effective teaching style*, as highlighted within the framework of *Self-Determination Theory* (SDT; Ryan and Deci, 2000, 2016; Codina et al., 2020; Dash et al., 2020). In short, this is the kind of teaching that minimizes stress factors, while *nonregulatory or dysregulatory teaching* is teaching that contributes to an increase in such stress factors. Consequently, it can be inferred that an increase in stress factors in the teaching and learning process would be a correlate of nonregulatory or dysregulatory teaching (de la Fuente et al., 2020e). This effect can be produced in a generalized way or in specific factors. In our study, a small increase in specific stress factors characteristic of *teaching methodology difficulties* and *heavy workload* was demonstrated. Concerning stress factors that originated in the teaching process, our results indicate that:

(1) Certain specific decisions in the teaching process were probably hasty and inadequate, causing distress and concern in the students; they did not fit into the design of the original subject syllabus and led to a perceived loss of control, an aspect that tends toward student stress (Goe et al., 2008; González-Cabanach et al., 2016, 2018). Previous research has demonstrated students’ emotional dependence on the teaching process and on interaction with the teacher (Mainhard et al., 2018);
(2) However, these mismatches were not determined by the academic year, rather, determinants of negative academic emotions and the state of burnout were stable despite COVID-19. For this reason, we argue that the teachers’ teaching styles remain stable despite the pandemic and seem to operate as a protective factor against stress (Codina et al., 2020; Dash et al., 2020).

Stress factors that emerge from the *learning process* are indirectly triggered through the teaching process (a predictive aspect that has been shown in the results) but are also triggered directly by the student. Prior evidence has shown that the student’s level of regulation determines their stress factors while learning (de la Fuente et al., 2020). Therefore, student levels of nonregulation and dysregulation would be accompanied by higher levels of stress factors while learning, thereby explaining the increase in the negative emotions of *boredom* in men (Goetz et al., 2014). In this case, there could be an increase in the stress factor of perceived loss of control over achievement (Cole and Sapp, 1988). Although this study did not test the level of regulation—neither general regulation nor regulated learning—certain difficulties or stress factors can be attributed to a lack of student regulation when facing this new situation (Wolff et al., 2020).

In this research, these are factors that directly cause negative academic emotions or burnout. According to Maslach and collaborators (Maslach and Jackson, 1981; Maslach and Leiter, 1997), the concept of *burnout* (Schaufeli et al., 2002; Schaufeli and Bakker, 2004; Salanova et al., 2010) has a three-dimensional structure with the components of exhaustion, cynicism, and loss of self-efficacy or competence. In line with the results found here, students with burnout were unable to adapt to situations of contextual stress, producing in them a sense of lack of energy (exhaustion), loss of interest in and value given to study (cynicism), and increased doubts about their capacity as students (loss or lack of efficacy). The negative repercussions of this syndrome on students’ health, learning, and well-being have been corroborated (Schaufeli et al., 2002).

However, the effect on engagement was minimal and very inconsistent. Given the influence from positive psychology (Seligman and Csikszentmihalyi, 2000) and interest in studying positive aspects of individuals as opposed to dysfunctional states, the present topic has been approached from a focus on the conceptual opposite of burnout, namely, engagement (Salanova et al., 2000; Maslach et al., 2001; Salanova, 2003; Schaufeli and Bakker, 2004; Kim et al., 2009). Engagement is a persistent positive, affective, and motivational state characterized by three dimensions: vigor, dedication, and absorption. Vigor, in contrast to exhaustion, involves a high level of energy and effort in the face of difficulties and setbacks. Dedication, in contrast to cynicism, is characterized by a high level of involvement and enthusiasm about the task; and absorption, as opposed to feelings of inadequacy, is associated with feelings of happiness and a high level of concentration on the task. Studies on engagement in the university setting and in the Spanish geographical context are few; more extensive work has been done in the United States, Canada, and Australia (Casuso-Holgado, 2011; Kahu, 2013; Maroco et al., 2016). As opposed to burnout, engagement is considered an indicator of subjective well-being, greater satisfaction, and lesser tendency toward dropout (2007; Salanova et al., 2005; Zhang et al., 2007; Maroco et al., 2016; Zhang and McNamara, 2018).

### Student Gender as a Modulator of Emotions and Academic Stress at University

As repeatedly presented in the results, students who identified as women scored higher on certain stress factors that were typical of inconsistencies in the teaching process but not due to dysregulatory changes in the academic year of the COVID-19 outbreak. This occurs both in partial factors of the *teaching process* (methodological difficulties, heavy workload) and in the *learning process* (loss of control over achievement). Women had higher scores in anxiety, which is consistent with prior evidence reporting gender differences in response to academic stress (Wilson et al., 1996; Giota and Gustafsson, 2020). Nonetheless, this explanation must be qualified because it is possible to make a case for the resilience of women (Yu and Chae, 2020) and greater self-regulation (Weis et al., 2013). Previous research has shown that women made greater use of problem-focused coping strategies, and men used more emotion-focused strategies (de la Fuente et al., 2013). In other words, their greater engagement with the teaching–learning process made them follow its demands more closely and be more sensitive to changes therein, especially if there were real incongruencies. Levels of students’ personal self-regulation have also been shown to be predicted by contexts lacking regulation (nonregulation or dysregulation) (de la Fuente et al., 2009). This is consistent with the women’s higher scores in *dedication* to task (a correlate of engagement) in the present pandemic situation, which could be interpreted as a higher level of self-regulation and desire to manage the incongruencies that the pandemic has brought about (Pomerantz et al., 2002). In conclusion, the response pattern of women is that of greater perception of specific stress factors in teaching and learning and a higher negative emotion of anxiety, but the pattern also revealed a greater persistence in terms of tasks, which is a behavior typical of engagement. The results of this study shed light on how the gender variable affects the relationship patterns between stress, burnout, and engagement, as the effects found previously have not been clear and results were contradictory. In some studies, men obtained higher scores than women in cynicism (Grau et al., 2000). Regarding engagement, certain studies reported that women showed higher levels in its three dimensions (Martínez and Salanova, 2003). Others underscore differences in dedication but not absorption (Durán et al., 2006), and there are studies where differences do not appear at all (Casuso-Holgado, 2011).

In the case of men, it is notable that all scores decreased. This drop is consistent with the increase in men’s negative emotions of boredom (deactivating emotion) and in the motivational state of cynicism, a typical burnout behavior (Wolff and Martarelli, 2020). Prior research has consistently shown that the emotions of enjoyment and boredom are inversely proportionate and inversely predict achievement, with boredom predicting poorer performance (Pekrun et al., 2010; Putwain et al., 2017; Obergriesser and Stoeger, 2020; Sharp et al., 2020). Consequently, boredom and cynicism seem to jointly reflect a manner of facing the situation with a lack of engagement, with passive avoidance or disconnection, thus leading to poorer performance and eventually to drop out from the teaching–learning process. We may therefore conclude that the incongruencies that characterize the COVID-19 context affect men in a different pattern, through loss of connection to the teaching–learning processes, manifest as a negative emotional state and avoidance motivation (Leonard et al., 2020; Pouratashi and Zamani, 2020). On the other hand, self-regulation and boredom have been shown to be inversely related, since a high level of self-regulation is associated with a low propensity to boredom (Isacescu et al., 2017; van Tilburg and Igou, 2017; Bieleke et al., 2020; Wolff et al., 2020). Moreover, boredom seems to be related to cognitive problems, and to physical and emotional self-regulation (Isacescu et al., 2017; Wolff et al., 2020). While high self-regulation is linked quite consistently with positive outcomes, a propensity to boredom is related mainly to negative outcomes (Eastwood et al., 2012).

From a complementary perspective, prior studies have found that the negative deactivating emotion of boredom has greater weight in class and study situations, while the positive deactivating emotion of relief is more relevant in exam situations. However, the relationship persists across class, study, and exam situations, revealing stability in the students’ emotional responses, according to their learning approaches: negative emotions are typical of surface approaches to learning, while positive emotions are typical of deep approaches (de la Fuente et al., 2020d, e). This would confer a personalistic component to learning approaches, that is, a stable motivational-affective, personal style (Zhang, 2003).

### Limitations and Future Lines of Research

One limitation of this study refers to the research methodology used, given that an ex post facto design itself limits the inferences that can be drawn. In addition, since we could not ensure that the academic subjects were the same ones each year, we cannot infer that methodological changes were owing exclusively to the COVID-19 crisis. Academic subjects were not grouped a priori according to regulatory, nonregulatory, or dysregulatory teaching systems, something which would have made it possible to attribute a cause to this factor, nor were students categorized as practicing self-regulated, nonregulated, or dysregulated learning. Nonetheless, recent research has documented this (de la Fuente et al., 2019, 2020). Future research should clarify these aspects, given that the pandemic continues to persist, and we will have the opportunity to analyze these variables. Other specific explanatory behaviors, such as rumination, were outside the scope of this study (Shaw et al., 2017; Kamijo and Yukawa, 2018).

Predictive and explanatory analysis of how different sources of stress affect academic emotions, and how these relate to the motivational states of burnout-engagement, has particular relevance for higher education institutions. Also significant is the role of gender as a modulating variable in the development of different stress profiles at university. This analysis can help identify which stressors in the teaching and learning process may affect university students’ stress levels and, consequently, how to reduce these levels (Cotton et al., 2002; Ketonena et al., 2019) and ensure a greater degree of academic success, student well-being, and lower dropout rates. However, these results should be viewed with caution, due to the gender ratio in the sample studied. Future studies could consider a few performance indexes (marks, exams passed, dropout rates, etc.) and teaching styles, in order to better contextualize these results. Because stress is the result of a relationship between the subject and their environment (Lazarus and Folkman, 1984), the present study has stressed the important role of regulatory teaching (Lekwa et al., 2018; de la Fuente et al., 2019, 2020a,c) within the relationship framework we examine.

### Implications for Psychoeducational Intervention

There are several implications for psychoeducational intervention. On one hand, teachers need to be trained in regulatory teaching processes; lack of regulation and improvised changes in activities and/or assessment during the teaching–learning process become stress factors for students (Freire et al., 2018). On the other hand, university students must be trained in self-regulated learning behaviors and made aware of the pitfalls of a lack of regulation or dysregulation in one’s own learning, especially in the case of younger men (Rubin et al., 2018).

It also seems reasonable to train students in stress management competencies for learning at university, given that unforeseen events can easily appear, as in the present situation. We should not assume that nothing will happen in the future; based on experience, it is possible that unexpected life events or academic incidents will occur, and we should be prepared to take them on in a resilient manner (Pozos-Radillo et al., 2014). It is the nature of life itself. In fact, prestigious universities and colleges in the US include the promotion of Physical and Psychological Health (avoiding unhealthy behaviors, having effective coping strategies for dealing with stress) in their educational objectives and mission statements (Oswald et al., 2004; Lipnevich et al., 2016). We also find a model to follow in certain Canadian universities, where a new line of research positions learning in the context of well-being (World Health Organization, 1986). Therefore, interventions to reduce burnout (and consequently dropout rates) should target emotions and emotion regulation.

Current events are forcing us to make broad behavioral adjustments in the organization of our personal, family, and academic life for the weeks ahead. To make these adjustments smoothly, we must keep in mind different behavioral principles and strategies for coping with the pandemic, something which is under-addressed in university Study Plans at every level. It is essential to design programs and improvement strategies for competency in dealing with the pandemic, from a psychological viewpoint (Robert, 2020) and from a psychoeducational viewpoint specifically. Some of these adjustment principles have already been formulated (de la Fuente et al., 2021). An example of specific strategies—already listed in another research report—include the following (de la Fuente, 2020):

#### On the Part of Teachers

(1) In the subjects you teach, maintain a *regulatory environmental design* that prompts a perception of control and continuity in students:
  a. Keep your usual contact hours with students, using appropriate technology media. Direct, online classes allow you to continue with the subject and lessen anxiety in the students.
  b. Make every adjustment so that all participants perceive normality and a sense of control. It is best to keep up the normal pace of the subject, although with adjustments as the situation requires. It is not a good time to make big, unexpected changes.
  c. If needed, adjust your assessment system and activities during this period. Make students aware that new situations involve new behavioral challenges and opportunities, for example, the chance to practice online teamwork from home.
(2) *Apply external regulation* to help students in their learning process:
  a. In case you have not already done so, this is a good time to convert all learning resources to digital formats and encourage students to learn autonomously from home. Keep this material and instructions well-structured because students are dealing with several modules at the same time.
  b. Plan regular, general messages and aids for your students, so they feel that the teaching–learning process continues with some normality.
  c. Offer personalized online consultation for students who need it. It is especially important to keep direct contact with the student representative in each class in order to be informed of any possible problems or help that students are needing.
  d. Regularly reevaluate whether students need adjustments to the material, assignments, etc.
  e. Pay attention to the emotional state and expectations of your students. Convey calm and assurance with your own behavior. Your students see themselves reflected in you, and in the image that you portray when interacting with them. Become a mentor that supports the process, also emotionally.

#### On the Part of Students

(1) *Self-regulate your own behavior*: while homebound, stick to your usual schedule.
  a. Following Circadian rhythm and keeping up personal habits go far to help maintain one’s sequence of activity, self-regulate, and not lose motivation.
  b. Give yourself daily doses of positive emotions and rewarding experiences while sheltering at home. It is very important to keep a positive emotional frame of mind. Distress (diffuse, negative emotionality, and discouragement) can be triggered by abrupt changes in one’s daily rhythm, or by a sense of uncertainty and loss of behavioral control.

1. *Self-regulate your learning behavior* during this period:
  a. Every day, plan objectives, schedules, and action steps, being flexible but also systematic.
  b. Exercise control over your behavior. Structure your continuous work time to include pauses for rest. Stop and take time for leisure activities (a substitute for outdoor activities). Tell yourself that you are doing the right thing. Use different relaxation techniques to decrease any anxiety.
  c. It is not a good time to take on complex issues in your life situation, because this may cause even greater stress and loss of situational control. If it is truly necessary, make small, gradual adjustments.
  d. Take advantage to catch up on matters that are pending, whether personal, family-related, or academic tasks. This is a gift of time.
  e. Reevaluate your daily behavior at the end of the day and redefine your objectives (family-related, personal, and academic) for the next few days.

## Data Availability Statement

The raw data supporting the conclusions of this article will be made available by the authors, without undue reservation.

## Ethics Statement

The studies involving human participants were reviewed and approved by Universidad de Navarra. The patients/participants provided their written informed consent to participate in this study.

## Author Contributions

JF: project IP, design, data analysis and initial writing. MP-B: bibliographic and text review. FS: data collection and review of the English text. MG-T, RA-G, FP-S, PP, and MG: data collection and final text review. All authors contributed to the article and approved the submitted version.

## Conflict of Interest

The authors declare that the research was conducted in the absence of any commercial or financial relationships that could be construed as a potential conflict of interest.
